# Influence of Eating Behavior and Dietary Patterns on Gut Microbiota Formation in Children with Autism Spectrum Disorder

**DOI:** 10.3390/nu18101506

**Published:** 2026-05-08

**Authors:** Natalia A. Smolko, Maria I. Markelova, Gulnaz E. Synbulatova, Dilyara R. Khusnutdinova, Albert A. Sufianov, Galina Z. Sufianova, Tatiana V. Grigoryeva, Albert A. Rizvanov, Rezeda A. Faizullina, Yana O. Mukhamedshina

**Affiliations:** 1 Ministry of Health of the Russian Federation, Kazan State Medical University, 420012 Kazan, Russia; sna997@yandex.ru (N.A.S.); r868@mail.ru (R.A.F.); 2 Kazan (Volga Region) Federal University, Institute of Fundamental Medicine and Biology, 420008 Kazan, Russia; mimarkelova@kpfu.ru (M.I.M.); gesynbulatova@kpfu.ru (G.E.S.); dilyahusn@gmail.com (D.R.K.); albert.rizvanov@kpfu.ru (A.A.R.); 3 Federal State Budgetary Scientific Institution “Russian Scientific Center of Surgery Named After Academician B.V. Petrovsky”, 119991 Moscow, Russia; sufianov@gmail.com; 4 The Research and Educational Institute of Neurosurgery, Peoples’ Friendship University of Russia (RUDN), 117198 Moscow, Russia; 5 Department of Pharmacology, Tyumen State Medical University, 625023 Tyumen, Russia; sufianova@tyumsmu.ru; 6 Department of Medical and Biological Sciences, Academy of Sciences of the Republic of Tatarstan, 420111 Kazan, Russia

**Keywords:** autism spectrum disorders, gut–microbiota–brain axis, dietary intervention

## Abstract

**Background/Objectives:** Autism spectrum disorder (ASD) is often associated with gastrointestinal dysfunction and gut microbiota alterations. This study aimed to characterize the gut microbiota in children with ASD in relation to nutritional factors and to evaluate the effects of dietary interventions combined with probiotics. **Methods:** The study included 96 children with ASD and 39 neurotypical controls. Follow-up data after intervention were available for 60 children with ASD. Gut microbiota composition was assessed by 16S rRNA sequencing, and fecal calprotectin and zonulin were measured before and after intervention. Most children with ASD (*n* = 91) received a rotational or elimination diet for six months, and all participants with ASD received probiotics for 1.5 months. **Results:** Children with ASD showed significant microbiota changes compared with controls, including increased *Prevotella*, *Sarcina*, NK4A214 group, and RF39 taxon, along with reduced butyrate-producing bacteria, such as *Roseburia*, *Eubacterium xylanophilum* group, and *Eubacterium ventriosum* group. Formula feeding was associated with increased *Odoribacter*, whereas food selectivity was linked to higher *Prevotella*, *Sarcina*, *Methanobrevibacter*, and RF39. A rotational diet increased *Erysipelotrichaceae* UCG-003 and *Streptococcus*, while an elimination diet increased *Butyricicoccus* and reduced fecal calprotectin (*p* = 0.023). Fecal zonulin decreased significantly after intervention in the follow-up ASD subgroup (*p* = 0.018). **Conclusions:** The obtained data suggest that children with ASD may exhibit certain microbiota features associated with nutritional patterns. Dietary interventions combined with probiotics appear to be associated with microbiota modulation and a tendency toward improvement in markers of intestinal inflammation and barrier function.

## 1. Introduction

Autism spectrum disorder (ASD) is currently an extremely common neurodevelopmental disorder. According to the latest international data, every 31st child aged 4–8 years has ASD [1]. The increasing statistics on ASD prevalence inevitably expand the pool of research aimed at identifying the causes, mechanisms, and correction methods for ASD. Scientific studies have established that ASD is accompanied not only by neuropsychiatric symptoms but also by a higher frequency of somatic pathologies. A feature of these somatic conditions is the complexity of their diagnosis, timely treatment, and prevention due to the specifics of the neuropsychiatric status of patients with ASD. According to research data, individuals with ASD are particularly at risk of immune dysregulation, more frequent organic and functional gastrointestinal pathologies, and eating disorders [2,3]. Somatic diseases can act both as a risk factor for the development of ASD and exacerbate its core symptoms. This influence is primarily mediated through the gut–microbiota–brain axis, which is one of the key pathways in the pathogenesis of ASD.

It has been established that the development and functioning of brain cells are closely linked to the features of the gut microbiota, the study of which is of particular importance [4,5]. It is known that gut dysbiosis occurs more frequently in children with ASD than in their neurotypical peers [6], and it contributes to the development of systemic inflammation through the activation of T–helper cells. These cells affect microglial activity and the integrity of the intestinal and blood–brain barriers, the permeability of which is increased in ASD [7]. One study found a higher relative abundance of *Bacteroides* and *Ruminococcus* in children with ASD compared to neurotypical children [8]. Another meta-analysis revealed increased levels of *Parabacteroides*, *Anaerostipes*, *Faecalibacterium*, *Clostridium*, *Dorea*, *Phascolarctobacterium*, *Lachnoclostridium*, *Catenibacterium*, and *Collinsella*, as well as decreased levels of *Barnesiella*, *Odoribacter*, *Paraprevotella*, *Blautia*, *Turicibacter*, *Lachnospira*, *Pseudomonas*, *Parasutterella*, *Haemophilus*, and *Bifidobacterium* in children with ASD [9]. The number of studies describing the metabolic activity of bacteria and the influence of their metabolites on the neural activity of the brain, which may be reflected in the clinical symptoms of ASD, is increasing [10,11].

As noted earlier, against the background of a higher prevalence of gastrointestinal pathologies and altered bacterial metabolic profiles, intestinal permeability may change [12]. This provokes the entry of various antigens into the bloodstream and leads to the development of systemic inflammation. The problem is exacerbated by the fact that gastrointestinal pathology can lead to additional sleep disturbances, irritability, and aggression, thereby worsening the core symptoms of ASD [13]. Since parents and some medical professionals may misinterpret aggressive or agitated behavior in children with ASD as core manifestations of the disorder rather than as indicators of gastrointestinal discomfort or pain, the diagnosis of gastrointestinal pathology and associated dysbiosis can be delayed. This is particularly challenging in children with limited or no verbal communication, who may react to pain in specific ways. Late verification of somatic pathology leads to a deterioration in the child’s health, chronicity of the process, and exacerbation of core ASD symptoms due to the impact on the gut–microbiota–brain axis. Therefore, studying the features of gut microbiota composition, the prevalence of comorbid pathologies, and their connection with the core symptoms of ASD is one of the most critical issues actively investigated in recent years.

The aim of this study was to investigate the gut microbiota in children with ASD, assessing indicators of intestinal permeability and inflammation. The work focused on identifying taxonomic features of the microbiota in various ASD subgroups, considering the influence of early nutritional history and current eating behavior patterns, as well as exploring the potential of personalized dietary recommendations and long-term probiotic intake to modulate the microbial community.

## 2. Materials and Methods

### 2.1. Subjects

The study involved 96 children diagnosed with ASD, aged 3–8 years (5.38 ± 2.59), including 81 (84.38%) boys and 15 (15.63%) girls. The control group (healthy children) consisted of 39 children aged 3–8 years (5.28 ± 1.58), including 19 boys (48.72%) and 20 girls (51.28%). Children with ASD were diagnosed at the “Republican Clinical Psychiatric Hospital named after Academician V.M. Bekhterev of the Ministry of Health of the Republic of Tatarstan”.

Inclusion criteria for children with ASD were age 3–8 years, a diagnosis of ASD according to the criteria of the International Classification of Diseases, 10th revision, and confirmation by the Autism Diagnostic Observation Schedule, second edition (ADOS-2). Inclusion criteria for children in the Control group: age 3–8 years, absence of an ASD diagnosis and any other psychiatric diagnosis.

Exclusion criteria for children in the ASD and Control groups were uniform and included: presence of concomitant chronic diseases, including allergic ones; hormonal/immunosuppressive therapy; use of antibacterial drugs and probiotics during the last month.

The children were comparable in terms of age; however, the ASD1 group was predominantly male, reflecting the known sex ratio of this disorder. Nevertheless, it is well established that no sex-related differences are present in childhood, and that differences in the gut microbiota between sexes begin to emerge only during puberty [14].

The conduct of the study was approved by the local Ethics Committee of the Kazan State Medical University of the Ministry of Health of the Russian Federation (extract from the meeting minutes No. 11 dated 19 December 2023). Legal representatives of the children provided informed voluntary consent to participate in the study.

### 2.2. Design of the Study and Sample Collection

**Figure 1:**
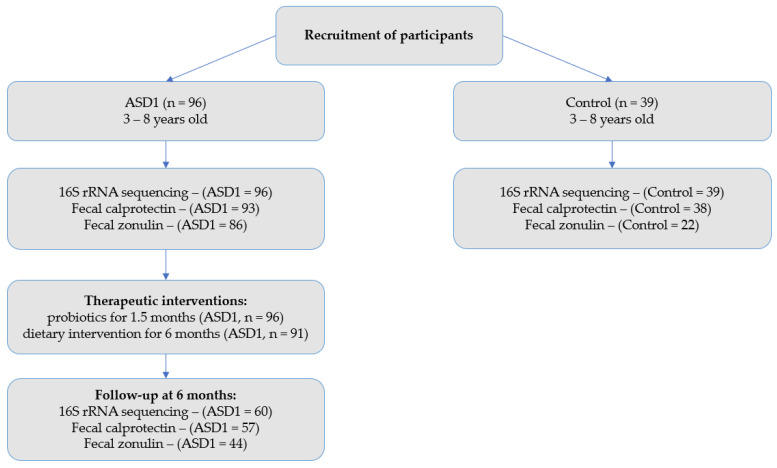
CONSORT-style flow diagram of the study.

The flow of participants through the study is shown in Figure 1.

The study design included anamnesis collection, questionnaire surveying, and stool sample collection at the beginning of the study (forming the ASD1 group, *n* = 96) and during follow-up after 6 months (forming the ASD2 group, *n* = 60, and the control group, *n* = 39), followed by gut microbiota composition analysis.

To assess dietary diversity, children were divided according to the number of food items consumed in their diet. According to some sources, selectivity is described as the consumption of fewer than 20 food items in the diet; accordingly, our classification was based on this aspect [15]. Clinical verification of data by a physician during the appointment was a mandatory stage of information collection, aimed at controlling the validity of parental responses. The diet of children with non-selective eating included a standard range of foods: dairy products (cottage cheese, milk, cheese, plain yogurt), meat (beef, chicken, turkey), white fish (rarely), cereals (buckwheat, rice, multigrain), pasta, vegetables (cucumbers, tomatoes, occasionally bell peppers), fruits (apples, bananas, pears), as well as sugary products and simple carbohydrates. In contrast, the diet of children with selective eating was characterized by a predominance of simple carbohydrates (sugar, refined flour products), reduced intake of fiber-rich vegetables and fruits, and low consumption of animal protein.

Stool samples were collected by the participants’ legal guardians at home and transported to the laboratory within two hours of collection. All samples were stored at −80 °C until DNA extraction.

### 2.3. Therapeutic Intervention in Children with ASD

The prescription of a rotational or elimination diet for children in the ASD1 group (*n* = 96) was based on clinical examination, parental interview, and laboratory assessment (total and specific IgE to food allergens) [16]. A total of 17 (17.7%) children were prescribed an elimination diet; this number included children who had been adhering to an elimination diet on the recommendation of doctors long before the start of the study. A total of 74 (77.1%) children were prescribed a rotational diet based on the results of the questionnaire survey, clinical examination, and laboratory assessment. Five (5.2%) children did not require any dietary intervention, as they lacked the clinical and diagnostic markers necessary for the prescription of any dietary intervention option. Of the 96 children with ASD at baseline, 60 completed the 6-month follow-up and fully adhered to the dietary recommendations. These 60 children constituted the ASD2 group, which included 47 (78.3%) children who followed a rotational diet and 13 (21.7%) children who followed an elimination diet. All children in the ASD1 group were prescribed a probiotic (Probiotics International Ltd., ADM Protexin Ltd.) for a period of 1.5 months, which contained *Lactobacillus casei PXN 37*, *Lactobacillus rhamnosus PXN 54*, *Streptococcus thermophilus PXN 66*, *Lactobacillus acidophilus PXN 35*, *Bifidobacterium breve PXN 25*, *Bifidobacterium infantis PXN 27*, and *Bifidobacterium longum PXN 30* at a quantity of 1 billion (1 × 10^9^) CFU/sachet, and a prebiotic: FOS (fructooligosaccharides) 990 mg. The long-term course of probiotics is supported by studies on their effectiveness in children with ASD [17,18].

It is worth noting that more than half of the children with ASD were taking dietary supplements (vitamin D, omega-3, magnesium). Furthermore, 6 (6.3%) of the children with ASD were receiving psychiatrist-prescribed medications (antipsychotics, antiepileptic agents); nevertheless, we deemed it appropriate to include these children in the study.

### 2.4. 16S rRNA Sequencing and Analysis of Stool Samples

Analysis of gut microbiota composition was performed using high-throughput 16S rRNA gene sequencing. DNA was extracted from fecal samples using the commercial kit “SKYAMP STOOL DNA”, art. EDC328, SkyGen (Beijing, China), according to the manufacturer’s instructions. Library preparation was carried out following the protocol “Preparing 16S Ribosomal RNA Gene Amplicons for the Illumina MiSeq System”.

The V3–V4 region of the 16S rRNA gene was amplified using the primers: forward 341f:5′-TCGTCGGCAGCGTCAGATGTGTATAAGAGACAGCCTACGGGNGGCWGCAG-3′; reverse 806r: 5′-GTCTCGTGGGCTCGGAGATGTGTATAAGAGACAGGACTACHVGGGTATCTAATCC-3′. Subsequently, the obtained PCR products were purified using AMPure XP (Beckman Coulter, Brea, CA, USA) magnetic beads according to the protocol. The resulting DNA libraries were sequenced on an Illumina MiSeq platform (in 2 × 300 mode) following the manufacturer’s protocol.

The obtained 16S rRNA gene sequences (reads) underwent sequencing quality control using FastQC software (ver. 0.12.0). Using Trimmomatic software (ver. 0.39) [19], low-quality reads as well as technical sequences (adapters, linkers, etc.) ligated during library preparation were removed. Filtered reads were analyzed using QIIME2 (version 2022.8) [20] and the DADA2 (ver. 1.20) algorithm [21]. Taxonomic profiling was performed using the latest version of the SILVA 138 database with a 99% similarity threshold [22].

To characterize the alpha diversity of the intestinal microbial community, the study of phylogenetic diversity, the Chao1 index, the Shannon index, the Simpson index, and the number of ASVs were used:

(1) The Chao1 index predicts the number of taxa in a sample by extrapolating rare microorganisms that may have been missed due to undersampling.
(2) Phylogenetic Diversity (PD) is a measure of diversity based on the evolutionary development of microorganisms. This indicator demonstrates the phylogenetic distance of microorganisms within the microbial community on the phylogenetic tree.
(3) The Shannon index is a measure of the diversity and evenness of taxa in a microbial community. An increase in the diversity of microorganisms and an increase in the evenness of species distribution across taxa increase the Shannon index, while the predominance of dominant species lowers the index.
(4) The Simpson index demonstrates the probability that two randomly selected individuals belong to different species.
(5) ASVs are unique DNA sequences obtained from high-precision sequencing of a metagenomic sample.

### 2.5. Fecal Markers of Intestinal Inflammation and Permeability

Fecal zonulin concentrations were determined using a competitive enzyme-linked immunosorbent assay (ELISA) (IDK^®^ Zonulin ELISA, Immundiagnostik AG, Bensheim, Germany; REF K5600) according to the manufacturer’s protocol in ASD1 (*n* = 86), ASD2 (*n* = 44) and Control (*n* = 22) groups. Paired measurements (both baseline and follow-up) were available for 42 children with ASD, of whom 33 were on a rotational diet. Stool extracts were incubated with a biotinylated zonulin tracer in microplate wells coated with polyclonal anti-zonulin antibodies. Free zonulin in the samples competed with the tracer for antibody binding. After washing, peroxidase-conjugated streptavidin and tetramethylbenzidine (TMB) substrate were added. The reaction was terminated with an acidic stop solution, and absorbance was measured spectrophotometrically. According to the manufacturer, the median fecal zonulin concentration in healthy individuals is 61 ± 46 ng/mL. Concentrations were defined as follows: <83.15 ng/mL, normal; 83.15–110.0 ng/mL, elevated; >110.0 ng/mL, high.

Fecal calprotectin concentrations were determined using a sandwich ELISA (Calprotectin ELISA, BÜHLMANN Laboratories AG, Schönenbuch, Switzerland; REF EK-CAL) according to the manufacturer’s instructions in ASD1 (*n* = 93), ASD2 (*n* = 57) and Control (*n* = 38) groups. Paired measurements (both baseline and follow-up) were available for 57 children with ASD, of whom 44 were on a rotational diet. Briefly, stool extracts were added to microplate wells coated with monoclonal anti-calprotectin antibodies; bound calprotectin was detected using an enzyme-labeled secondary antibody, followed by substrate development and spectrophotometric measurement of absorbance. According to the manufacturer, reference values were defined as follows: <80 μg/g, normal; 80–160 μg/g, borderline; >160 μg/g, elevated.

### 2.6. Statistical Analysis

Statistical calculations were performed using the R programming language, version 4.5.0, within the RStudio environment, version 2025.05.0. The normality of distribution for all variables was assessed using the Shapiro–Wilk test. The vast majority of the investigated parameters exhibited a non-normal distribution.

Statistically significant differences in these parameters between the study groups were identified using the Kruskal–Wallis test with post hoc Dunn’s test, followed by the application of the Benjamini–Hochberg correction for multiple comparisons. Correlation analysis was conducted using Spearman’s rank correlation coefficient. Correlation strengths were interpreted as follows: moderate (0.3–0.5), noticeable (0.5–0.7), high (0.7–0.9), and very high (0.9–0.99). A *p*-value of <0.05 was considered the critical level of significance.

For the comparison of paired (related) samples with ordinal, non-normally distributed data, the Wilcoxon signed-rank test was used. For the comparison of categorical variables between two independent groups, Fisher’s exact test was used. A *p*-value < 0.05 was considered statistically significant.

## 3. Results

### 3.1. Gut Microbiota Composition and Nutritional Features in Children with ASD Before Therapeutic Intervention

#### 3.1.1. Gut Microbiota and Infant Feeding in Children with ASD During the First Year of Life

Analysis of the prevalence of different feeding types during the first year of life revealed no statistically significant differences between the ASD and control groups (Table 1).

We also took into account the first complementary feeding products that were introduced to children during their first year of life, as well as the children’s reactions to the first complementary food product. It was found that in the ASD1 group (*n* = 96), the mean age of complementary feeding introduction was 5.81 (±1.77) months, while in the Control group (*n* = 39), it was 5.63 (±1.09) months; the differences were not statistically significant (*p* = 0.949). No differences in the choice of the first complementary food product were observed either (Supplementary Table S1). The analysis showed that among children in the ASD1 group (*n* = 96), 81 children (84.4%) had a positive reaction to complementary feeding, while 15 children (15.6%) had a negative reaction. In the Control group (*n* = 39), a positive reaction was observed in 36 children (92.3%), and a negative reaction in 3 children (7.7%); however, the differences did not reach statistical significance (*p* = 0.247).

**Figure 2:**
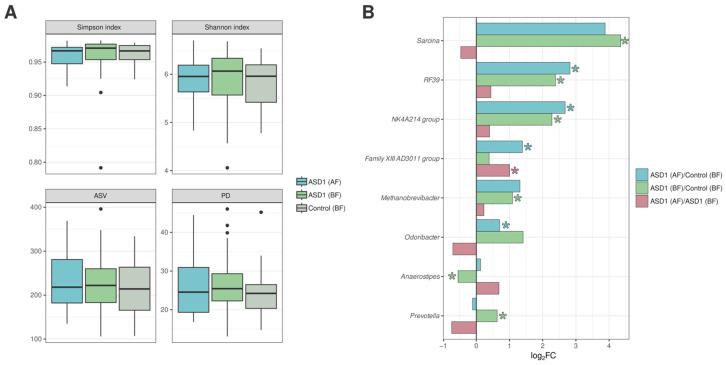
(**A**) Alpha-diversity indices. ASD1 (FF): children with ASD on formula feeding; ASD1 (BF): children with ASD on breastfeeding; Control (BF): typically developing children on breastfeeding. (**B**) Taxa showing statistically significant differences in relative abundance between comparison groups. Results are presented as log_2_ fold change, calculated as the ratio of the mean relative abundance of taxa in the compared groups. The asterisk (*) indicates statistically significant differences (*p* < 0.05).

We compared the gut microbiota of children with ASD who were breastfed (BF, *n* = 61) or formula-fed (FF, *n* = 25) during the first year of life, as well as breastfed children from the control group (BF, *n* = 31). No statistically significant differences in alpha diversity were found between the study groups (Figure 2A).

In the ASD1 (BF) group compared to the control (BF) group, there was a significant increase in the levels of *Prevotella* (*p* = 0.011), *Sarcina* (*p* = 0.023), the NK4A214 group (*p* = 0.025), RF39 (*p* = 0.021), and *Methanobrevibacter* (*p* = 0.016). Concurrently, a decrease in the abundance of *Anaerostipes* (*p* = 0.041) was observed. In the ASD1 (FF) group compared to the control (BF) group, significantly higher levels of *Odoribacter* (*p* = 0.030), the NK4A214 group (*p* = 0.002), RF39, and the Family XIII AD3011 group were found (Figure 2B).

Comparison between the ASD1 (FF) and ASD1 (BF) groups also revealed significant differences. Formula feeding in the ASD1 group during the first year of life was associated with an increase in the Family XIII AD3011 group (*p* = 0.032).

#### 3.1.2. Gut Microbiota and Dietary Patterns at the Time of the Study

According to the results of our study, children in the ASD1 group (*n* = 61, 63.5%) had a significantly higher occurrence of food selectivity compared to the control group (*n* = 8, 20.5%; *p* < 0.001). Analysis of the relationship between food selectivity and feeding type during the first year of life in the ASD1 group revealed no statistically significant differences (*p* = 0.329). The likelihood of developing food selectivity in the ASD1 (BF) group was 1.79 times lower than in the ASD1 (FF) group, but the difference was not statistically significant (OR = 0.56; 95% CI: 0.20–1.54; Supplementary Table S2).

**Figure 3:**
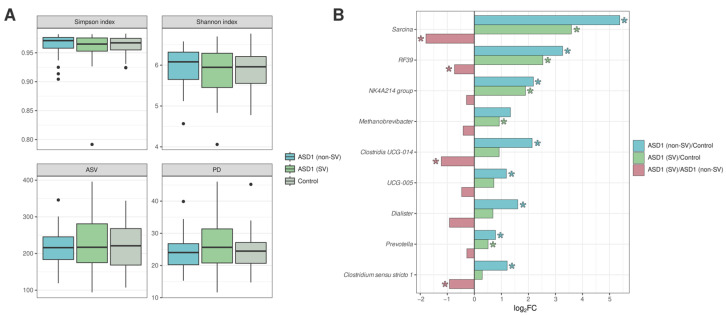
(**A**) Alpha-diversity indices. ASD1 (non-SV): children with ASD without selective eating patterns; ASD1 (SV): children with ASD with selective eating patterns; Control: typically developing children from the control group. (**B**) Taxa showing statistically significant differences in relative abundance between comparison groups. Results are presented as log_2_ fold change, calculated as the ratio of the mean relative abundance of taxa in the compared groups. The asterisk (*) indicates statistically significant differences (*p* < 0.05).

Comparison of gut microbiota based on the presence of food selectivity showed no significant differences in alpha diversity (Figure 3A).

However, the ASD1 group with selective eating (ASD1 (SV), *n* = 61) differed from the control group by an increase in *Prevotella* (*p* = 0.035), the NK4A214 group (*p* = 0.013), *Sarcina* (*p* = 0.049), *Methanobrevibacter* (*p* = 0.021), and RF39 (*p* = 0.026). The ASD1 group without selective eating (ASD1 (non-SV), *n* = 35) also exhibited dysbiosis, with an increase in *Prevotella* (*p* = 0.002), *Sarcina* (*p* = 0.001), the NK4A214 group (*p* = 0.005), RF39 (*p* < 0.001), as well as *Dialister* (*p* = 0.048) and *Clostridia* UCG-014 (*p* = 0.003). The latter taxon was negatively correlated with selectivity (r = −0.319). Direct comparison of the ASD1 (SV) and ASD1 (non-SV) groups showed an increase in RF39 (*p* = 0.008), *Clostridium* sensu stricto 1 (*p* = 0.030), and UCG-005 (family Oscillospiraceae) (*p* = 0.034) in the non-selective group (Figure 3B).

Given the influence of dietary patterns on dysbiosis severity, attention was paid not only to food selectivity but also to other forms of dietary restrictions. Among the 96 children in the ASD1 group, 83 (86.5%) did not follow any diet, while 13 (13.5%) followed an elimination diet. Among the latter, 11 children had been following an elimination diet on the recommendation of doctors long before the start of the study, and two children followed it based on their own beliefs about its effectiveness. Among the children on elimination diets (*n* = 13), nine (69.2%) followed a combined gluten-free/casein-free diet, three (23.1%) followed a gluten-free diet, and one (7.7%) followed a casein-free diet. For further analysis, children with ASD on various elimination diets were combined into a single subgroup (*n* = 13) to achieve higher statistical power.

Comparison of the ASD1 group on elimination diets (*n* = 13) with the ASD1 group not following any dietary restrictions (*n* = 83) revealed significant differences in microbiota composition (Table 2).

The ASD1 group without dietary restrictions (*n* = 83) showed a significant increase in *Ruminococcus* (*p* = 0.044), *Turicibacter* (*p* = 0.041), and *Erysipelatoclostridium* (*p* = 0.031). The ASD1 group following elimination diets (*n* = 13) was characterized by significantly higher levels of *Butyricicoccus* (*p* = 0.016) and the UCG-003 group (family Oscillospiraceae) (*p* = 0.023). Correlation analysis revealed that more frequent trying of new foods was significantly correlated with the level of *Flavonifractor* (r = −0.314).

### 3.2. Gut Microbiota Composition in Children with ASD During Therapeutic Intervention

Comparing the abundance of microbial genera by sex within the ASD and control groups revealed sex-related differences in several low-abundance taxa, with median relative abundance not exceeding 0.1% in any group. Importantly, none of these taxa showed a difference in abundance between the ASD and control groups (Supplementary Table S3).

**Figure 4:**
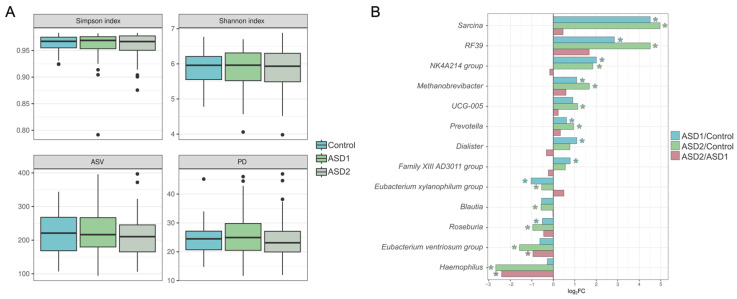
(**A**) Alpha-diversity indices. (**B**) Taxa showing statistically significant differences in relative abundance between comparison groups. Results are presented as log_2_ fold change, calculated as the ratio of the mean relative abundance of taxa in the compared groups. The asterisk (*) indicates statistically significant differences (*p* < 0.05).

Analysis of gut microbiota composition revealed characteristic dysbiotic shifts in the ASD groups (Figure 4).

In the ASD1 group (*n* = 96) compared to the control group (*n* = 39), there was a statistically significant increase in *Prevotella* (*p* = 0.009), *Dialister* (*p* = 0.028), the NK4A214 group (*p* = 0.002), *Sarcina* (*p* = 0.005), *Methanobrevibacter* (*p* = 0.043), and RF39 (*p* = 0.001). A decrease in the ASD1 group was recorded for *Roseburia* (*p* = 0.032) and the *Eubacterium* xylanophilum group (*p* = 0.015).

In the ASD2 group (*n* = 60) compared to the control group, an increase persisted for *Prevotella* (*p* = 0.014), the NK4A214 group (*p* = 0.003), *Sarcina* (*p* = 0.008), *Methanobrevibacter* (*p* = 0.026), and RF39 (*p* = 0.001). Additionally, an increase in UCG-005 (family Oscillospiraceae) (*p* = 0.033) was detected in ASD2. A decrease in this group was noted for *Roseburia* (*p* = 0.006), *Blautia* (*p* = 0.021), *Haemophilus* (*p* = 0.031), the *Eubacterium* ventriosum group (*p* < 0.001), and the *Eubacterium* xylanophilum group (*p* = 0.024).

Comparison between the ASD1 (*n* = 96) and ASD2 (*n* = 60) groups revealed only a dynamic decrease in the *Eubacterium* ventriosum group (*p* = 0.001) and *Haemophilus* (*p* = 0.049) in the ASD2 group.

Since the main dietary intervention was the rotational diet, we performed a paired comparison of patients in the ASD1 (*n* = 47) and ASD2 (*n* = 47) groups who fully adhered to the dietary recommendations. In the ASD2 group, the levels of *Monoglobus* and the *Eubacterium* ventriosum group significantly decreased, while *Sutterella*, *Butyricimonas*, and the *Lachnospiraceae* ND3007 group increased. No statistically significant differences were found in alpha diversity indices (Shannon index, Simpson index, number of ASVs, phylogenetic diversity, Chao1 index; *p* > 0.05 for all comparisons) (Table 3).

Comparison of gut microbiota in ASD2 patients on a rotational diet (*n* = 47) versus ASD2 patients on an elimination diet (*n* = 13) revealed statistically significant differences in the relative abundance of several taxa, with no significant differences in alpha diversity indices (*p* > 0.05) (Table 4).

The rotational diet group (*n* = 47) showed an increase in *Erysipelotrichaceae* UCG-003 (*p* = 0.029) and *Streptococcus* (*p* = 0.022). The elimination diet group (*n* = 13) showed an increase in *Phascolarctobacterium* (*p* = 0.041), *Lachnospiraceae* UCG-001 (*p* = 0.010), and CAG-56 (*p* = 0.018).

Effect sizes for group comparisons by feeding type and food selectivity ranged from 0.015 to 0.034, indicating small effects (Supplementary Table S4). Such small effect sizes are typical for microbiome studies, and despite the compositional nature of metagenomic data, our approach is standard and widely used in the field.

### 3.3. Assessment of Intestinal Inflammation and Permeability Markers in Patients with ASD

Comparative analysis of the ASD1, ASD2, and control groups revealed no statistically significant intergroup differences in fecal zonulin or calprotectin levels (Supplementary Table S5). The analysis also did not reveal any significant differences in the distribution of children according to the levels of intestinal inflammation markers depending on the normal/elevated/high categories (Supplementary Table S6). When comparing ASD1 subgroups based on adherence to special diets at the time of study enrollment, lower fecal zonulin levels were observed in children following elimination diets (Table 5).

**Figure 5:**
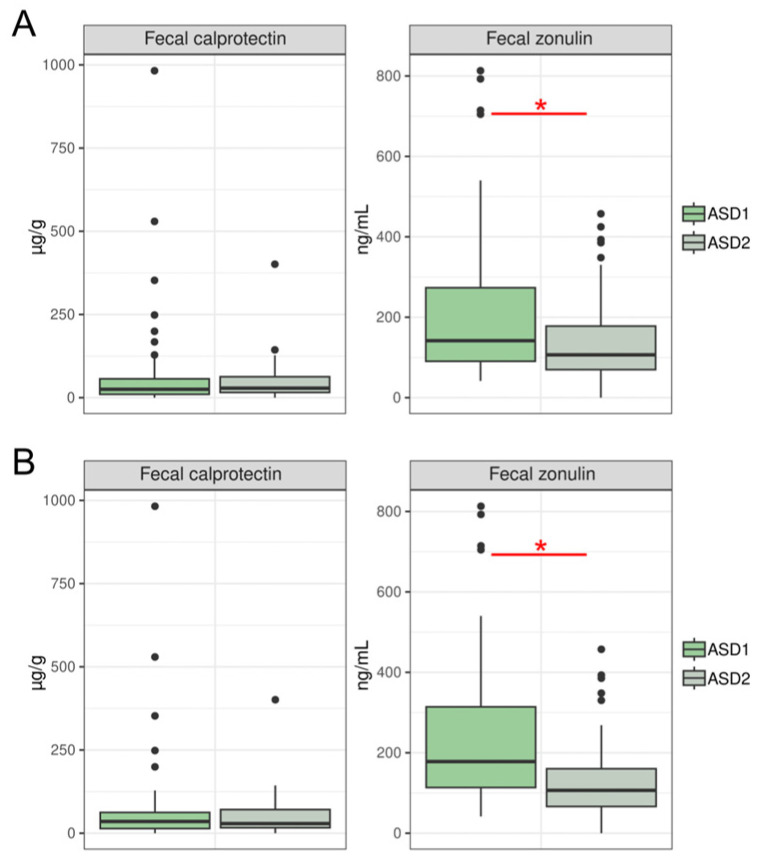
(**A**) Intestinal permeability markers in children with ASD before (ASD1, *n* = 42 for zonulin, *n* = 57 for calprotectin) and after (ASD2, *n* = 42 for zonulin, *n* = 57 for calprotectin) intervention. Data are presented as median [interquartile range]. Fecal zonulin: *p* = 0.018 *; fecal calprotectin: *p* = 0.657. (**B**) Intestinal permeability markers in children with ASD on a rotational diet before (ASD1, *n* = 33 for zonulin, *n* = 44 for calprotectin) and after (ASD2, *n* = 33 for zonulin, *n* = 44 for calprotectin) intervention. Data are presented as median [interquartile range]. Fecal zonulin: *p* = 0.001 *; fecal calprotectin: *p* = 0.763.

Paired comparison of ASD1 and ASD2 samples revealed significant differences. In the ASD2 group, fecal zonulin levels significantly decreased compared to ASD1 (Figure 5A).

A significant decrease in fecal zonulin levels in the ASD2 group was also found in paired comparisons of ASD groups on a rotational diet (Figure 5B).

Correlation analysis also revealed statistically significant relationships between taxonomic composition of the microbiota and markers of intestinal inflammation (*p* < 0.05). In the ASD1 group (*n* = 96), a positive correlation was found between fecal calprotectin levels and bacteria of the genus *Dorea* (r = 0.318) and *Streptococcus* (r = 0.394), as well as a negative correlation with the uncultured group UCG-003 (order Oscillospirales) (r = −0.322). Additionally, a positive correlation was found between fecal zonulin and *Dorea* (r = 0.323) and *Streptococcus* (r = 0.392) in the ASD1 group (*n* = 96).

## 4. Discussion

Nutrition is one of the main factors influencing the formation and composition of the gut microbiota [23]. From birth, the type of feeding is associated with which bacteria will predominate among the wide diversity of microbiota representatives [24,25]. Already during the first year of life, differences in nutrition were linked to the direction of gut microbiome maturation, which may have subsequently influenced its composition and functional role. The differences observed in the ASD1 (BF) group compared to the control (BF) group had a clinical explanation. Increased levels of *Prevotella* and *Sarcina* may contribute to the pathogenesis of gastrointestinal and neuropsychiatric disorders [26,27,28]. The clinical effects of increased *Methanobrevibacter* are associated with autism-like behavior in mouse models [29]. The decreased abundance of *Anaerostipes*, a genus that may exhibit both protective and adverse effects [30], may point to its dual role in interpretation. Thus, the microbiome profile of breastfed children with ASD combines features that may potentially contribute to both gastrointestinal dysfunction (through increased *Sarcina*) and modulation of neuropsychiatric disorders (through changes in *Prevotella* and *Anaerostipes*), highlighting the complex role of dysbiosis in the pathophysiology of the disorder.

In the ASD1 (FF) group compared to the control (BF) group, the increase in *Odoribacter* (*p* = 0.030) contradicts known data on the reduction in this genus in patients with cognitive impairment [31]. At the same time, the higher level of the NK4A214 group may be compensatory due to the production of short-chain fatty acids (SCFAs) [32]. The increase in the RF39 taxon, according to the literature, reflected a positive association with its higher prevalence among patients with severe forms of multiple sclerosis [33]. The increase in the Family XIII AD3011 group in the ASD1 (FF) group when comparing ASD1 (FF) vs. control and ASD1 (FF) vs. ASD1 (BF) also has a clinical interpretation. The literature describes an increase in the Family XIII AD3011 group in depressive states [34]; at the same time, recent data demonstrate beneficial effects of this phylogenetic group on intestinal permeability [35], which can likely be linked to its compensatory role. Thus, in our study, the microbiota of formula-fed children with ASD was characterized by an increased proportion of potentially unfavorable taxa. The taxonomic shifts presented demonstrate different patterns of microbiota profile formation depending on the type of feeding. However, despite the universally recognized benefits of breastfeeding, pronounced dysbiotic changes were also identified in the ASD1 (BF) group, suggesting that dysbiosis may not be prevented solely by the type of feeding.

In addition to the influence of feeding type on the gut microbiota composition of children with ASD, eating behavior is a critical factor that also determines the characteristics of the microbial taxonomic profile. As noted earlier, food selectivity is one of the most fundamental symptoms of eating disorders in children with ASD [36,37]. The presence of restrictive eating may be associated with the characteristics of neuropsychiatric development and also poses a serious problem for the normal socialization of children [38]. Selectivity is associated with inadequate intake of nutrients, protein, dietary fiber, and vitamins. Restriction of food groups that ensure gut microbiota balance, such as fruits and vegetables, as well as fiber from certain grains, may be associated with gut dysbiosis and an increase in pathogenic microbiota representatives [39].

In the selective eating group ASD1 (SV) compared to the control group, the detected significant shifts may point to a pathological transformation of the microbial profile toward an increase in pro-inflammatory taxa affecting the manifestations of gastrointestinal pathologies (*Prevotella*, *Sarcina*) as well as those associated with neuropsychiatric symptoms (*Prevotella*, *Methanobrevibacter*) [26,27,28,29]. At the same time, the increase in the NK4A214 group in the ASD1 (SV) group can be considered an adaptive role due to the activation of anti-inflammatory functions [32].

In the non-selective eating group, ASD1 (non-SV), compared to the control group, a persistent increase in the pro-inflammatory taxa *Prevotella* and *Sarcina* was maintained. In our study, we recorded a statistically significant increase in *Dialister*, whereas the literature describes its decrease in children with ASD [40]. This discrepancy is likely due to differences in the studied cohorts (China vs. Russia) as well as to the nature of eating behavior, since in our study the increase in *Dialister* was observed in the ASD1 (non-SV) group, possibly reflecting the influence of diet on the level of this taxon. The increase in the NK4A214 group and *Clostridia* UCG-014, the latter of which has a beneficial effect on cognitive functions, in the ASD1 (non-SV) group can be interpreted as a compensatory response of the microbiota to dysbiosis [41,42]. The negative correlation of *Clostridia* UCG-014 with food selectivity (r = −0.319) suggests a positive role of this taxon with respect to eating behavior. Accordingly, despite the diverse diet of children with ASD in this group, dysbiotic disturbances demonstrated persistent taxonomic differences when compared with the control group.

In the ASD1 (non-SV) group compared to the ASD1 (SV) group, an increase was found in the taxon with pathological characteristics RF39 [33], as well as in *Clostridium* sensu stricto 1 and UCG-005 (family Oscillospiraceae), which play a significant role in SCFA production [32,43,44], indicating a particular pattern of microbial changes with a compensatory increase in anti-inflammatory taxa. Accordingly, the gut microbiota profile depending on food selectivity is characterized by complex rearrangements with an increase in both pro-inflammatory and compensatory anti-inflammatory taxa.

A critical aspect that may also influence the composition of the gut microbiota is adherence to various types of diets, which is highly prevalent among children with ASD, with elimination diets being particularly popular [45,46]. Elimination diets, such as the gluten-free and/or casein-free diet, are aimed at reducing gastrointestinal symptoms (abdominal pain, constipation, diarrhea, etc.), normalizing microbiota composition, improving cognitive functions and sleep quality, although data on the effectiveness of these diets are controversial [47,48]. In our study, adherence to an elimination diet was associated with the features of the taxonomic composition of the gut microbiota. Thus, in ASD1 patients not adhering to any diet, the observed increase in the levels of *Ruminococcus* and *Turicibacter* (SCFA producers) contradicts known data on the reduction in these bacteria in patients with psychiatric disorders, including ASD [49,50]. This contradiction may indicate functional heterogeneity of the genus or specific features of the metabolic status in ASD without dietary correction. It is possible that the increase in the pool of SCFA producers in the ASD1 group without special dietary restrictions is a compensatory characteristic of the microbial profile of this patient group. The increase in *Erysipelatoclostridium* has been associated in the literature with depressive states [51], highlighting the particular nature of dysbiotic disturbances in this group.

In the group of ASD1 patients who followed an elimination diet at the time of enrollment in the study, the increase in Butyricicoccus may be clinically relevant, as this genus is considered an important butyrate producer whose levels are often reduced in several neurological diseases [52]. The increase in *Erysipelotrichaceae* UCG-003, a component of the normal microbiota of neurotypical children [53], also indicates potentially beneficial changes in microbial metabolism in the group of children with ASD on elimination diets. Accordingly, in our study, adherence to an elimination diet was associated with shifts toward an increased proportion of taxa that may exert positive clinical effects.

Since the prescription of a diet was one of the main corrective measures, the main types of dietary intervention were the rotational and elimination diets. The rotational diet, prescribed to the majority of patients in the ASD1 group, is a gentle method of dietary correction. Its principle is to consume foods from one group no more than once every 3–4 days and to reduce the antigenic load on the body, which, according to the literature, may positively affect the state of the intestinal barrier and its permeability [11,54,55]. The results of our analysis suggest that different types of dietary interventions (rotational and elimination diets) were associated with clinically significant shifts in the gut microbiota composition of ASD2 patients, which may reflect different mechanisms of their modulating effect on the microbiome.

The increase in *Erysipelotrichaceae* UCG-003 in the ASD2 group on a rotational diet may indicate a positive restructuring of the microbial community, since this taxon is considered a component of beneficial microbiota more characteristic of neurotypical individuals [53]. The increase in *Streptococcus*, on the contrary, is more often associated with pro-inflammatory effects and has been observed in patients with ASD [56]; however, its association with diet remains unclear and requires further study.

In the ASD2 group on an elimination diet, the increase in *Phascolarctobacterium* is consistent with data on its high abundance in ASD [56]; according to some reports, this taxon may contribute to the formation of autism-like behavior through propionate production [57], although its association with diet remains ambiguous. The increase in *Lachnospiraceae* UCG-001 differs from data obtained in elderly individuals with impulsive behavior, where this taxon was reduced [58], which may indicate an age-specific or nosological specificity of its role in children with ASD. The functional significance of CAG-56 in the context of dietary therapy requires further elucidation. Thus, different types of dietary interventions (rotational and elimination diets) are associated with multidirectional shifts in gut microbiota composition, affecting both potentially beneficial and potentially pathogenic taxa, highlighting the need for further studies in larger cohorts.

Since the main dietary correction intervention was the rotational diet, the paired comparison of the ASD1 and ASD2 groups (*n* = 47) according to this criterion also determined the characteristic features of gut microbiota restructuring. The decrease in *Monoglobus* is consistent with literature data on a reduction in their titer with positive dynamics of cognitive functions [59]. The decrease in the *Eubacterium ventriosum* group contrasts with its generally positive characteristics regarding SCFA production [60], which may be due to a restructuring of the microbial community toward other SCFA producers or to individual features of the metabolic activity of this taxon in the studied cohort. The increase in *Sutterella*, despite its association with neuroinflammation, may reflect adaptive activation of immunomodulatory mechanisms [61] or be a consequence of targeted dietary effects on specific strains. The increase in the relative abundance of *Butyricimonas* could be viewed as a favorable trend in microbiota restructuring, since its decrease has been observed in patients with cognitive impairment [31]. The increase in the *Lachnospiraceae* ND3007 group, representatives of which possess anti-inflammatory and butyrate-producing potential [62], characterizes the positive nature of the dynamic changes in the microbiota during the rotational diet. The obtained data suggest that during a comprehensive intervention with a leading role of the rotational diet, multidirectional shifts occur, combining both potentially positive and ambiguous changes, which may reflect the complexity of the mechanisms by which diet affects the taxonomic composition of the gut microbiota.

In addition to studying the complex relationships between gut microbiota and nutritional and eating behavior patterns in children with ASD, a generalized analysis of the taxonomic composition of the microbiota of the studied groups (ASD1, ASD2, control) was performed, which revealed no statistically significant differences in alpha diversity indices between the groups (*p* > 0.05). However, significant changes in the relative abundance of several bacterial taxa were identified, which correlate with the clinical presentation of ASD. Thus, the increase in *Prevotella* and *Sarcina* in both ASD groups is consistent with their role in the development of intestinal inflammation and gastrointestinal symptoms [26,27,28], confirming their potential contribution to the pathophysiology of ASD through the gut–microbiota–brain axis. *Methanobrevibacter*, an increase in which is associated with autism-like behavior in animal models [29], remained at high levels in both groups, which may suggest its potential role as a marker of the underlying disease. The increase in *Dialister* in the ASD1 group diverges from data on its decrease in ASD [40], indicating possible differences in the geographical characteristics of the study cohort and requiring further research on this genus. The limited information on the RF39 taxon, associated with severe forms of autoimmune pathology [33], also requires additional investigation in the context of ASD. The persistent increase in the NK4A214 group in both ASD groups, as well as the increase in UCG-005 (family Oscillospiraceae) in the ASD2 group, both of which are involved in SCFA synthesis [32,43], may be adaptive in the context of overall dysbiosis.

A decrease in the key SCFA producers *Roseburia* and the *Eubacterium xylanophilum* group was also recorded in the ASD1 and ASD2 groups compared to the control group [63,64,65,66], which may reflect pathological shifts in the microbial profile in ASD. A similar isolated decrease in the *Eubacterium ventriosum* group [60] and *Blautia* in the ASD2 group compared to the control group confirms a deficit of butyrate-producing bacteria [67]. The decrease in *Haemophilus* in the ASD2 group compared to the control group and in the dynamic comparison with the ASD1 group competes with data on its increase in ASD [68], which may reflect population-specific or dietary features of the studied cohort and requires further investigation. Thus, in the ASD1 and ASD2 groups after the interventions, common features of dysbiosis persist, manifested by an increase in pro-inflammatory and opportunistic taxa (*Prevotella*, *Sarcina*, NK4A214 group, RF39) and a decrease in butyrate-producing bacteria (*Roseburia*, *Eubacterium xylanophilum* group), while the few changes between the ASD groups indicate the persistence of dysbiotic disturbances and require further research.

Various dysbiotic changes in the microbiota create prerequisites for various disorders: they provoke gastrointestinal discomfort, contribute to the development of allergies and metabolic disorders [69,70]. Against the background of pathological conditions, intestinal permeability increases, which contributes to an imbalance in the microbiota–gut–brain axis. Fecal calprotectin (FC) and fecal zonulin (FZ) are among the accessible non-invasive markers of intestinal permeability. High levels of FC demonstrate increased permeability in the colon, while FZ does so in the small intestine. According to the literature, children with ASD more often have increased intestinal permeability compared to neurotypical children. This, in turn, facilitates the entry of various antigens, toxins, and signaling molecules into the systemic circulation, which sustain systemic inflammation and can negatively affect the blood–brain barrier [71,72].

Comparative analysis of the ASD1, ASD2, and control groups revealed no statistically significant differences between the compared groups (*p* > 0.05), which may be due to the variability of the obtained indicators, the heterogeneity of the cohort (not all children with ASD have increased intestinal permeability markers), and the influence of food selectivity, which may reduce FC and FZ concentrations. Also, according to the literature, FC levels in children with ASD may remain within reference values even in the presence of histological changes in the intestinal mucosa [73]. Paired comparison revealed a statistically significant decrease in fecal zonulin levels in the ASD2 group compared to ASD1, which may indicate a positive trend in the state of the intestinal barrier. These data are consistent with a possible association of the comprehensive correction (rotational diet plus probiotics) with normalization of small intestinal permeability [74,75]. However, due to the non-randomized design of our study, causal conclusions cannot be drawn.

The lower FC levels in children who followed elimination diets at the time of enrollment in the study, compared to children with ASD who did not follow any dietary restrictions, suggest an association between elimination diets and lower intestinal permeability. Similar results have been demonstrated in studies on the effectiveness of elimination diets in reducing fecal calprotectin in children with cow’s milk protein allergy [76], as well as in children with ASD using gluten-free and casein-free diets [71].

The statistically significant decrease in FZ levels in the ASD2 group in paired comparisons during the rotational diet can be viewed as an observed positive association with the diet, which may affect intestinal permeability and, possibly, the integrity of the "gut–microbiota–brain axis". Although there are no direct data on the study of intestinal inflammation markers during a rotational diet, there are studies describing a decrease in FZ in the ASD group on a balanced diet [77,78].

The identified correlations between markers of intestinal inflammation (FC and FZ) and bacteria of the genera *Dorea* and *Streptococcus* correspond to literature data on their high prevalence in ASD and their potentiating effect on intestinal mucosal inflammation [56,79,80]. The negative correlation of the UCG-003 taxon (family Oscillospiraceae) is consistent with its positive [81]. Thus, the obtained data confirm the close relationship between the dysbiotic composition of the microbiota and the state of the intestinal barrier in ASD.

### Study Limitations

The formation of a homogeneous sample of children with ASD in microbiome studies is substantially hindered by the pronounced clinical and behavioral heterogeneity of the disorder, high variability in dietary patterns, medication burden, and the significant influence of early and environmental factors [80,82]. In the present study, we undertook a number of measures aimed at minimizing these limitations, including strict inclusion and exclusion criteria, standardization of sample collection and processing protocols, and consideration of key clinical-demographic and behavioral factors during data analysis. Nevertheless, it is not possible to completely eliminate the influence of these sources of variability, which should be taken into account when interpreting and generalizing the obtained results. It should be noted that in our study, the dietary intervention and the probiotic course were not randomized. Although sex differences in the gut microbiota become significant only during puberty, residual confounding resulting from the pronounced sex imbalance between groups cannot be fully ruled out. It is impossible to disentangle the individual contribution of the probiotic and the diet when interpreting the results. Since the majority of children were taking dietary supplements (vitamin D, omega-3, magnesium), excluding them would have prevented us from carrying out the study with an adequately large patient cohort. Furthermore, the sample size of children with ASD and the control group was small. Large-scale, randomized controlled trials are necessary to confirm the associations established in our study and to isolate the specific effects of the diet and probiotic supplementation. One of the limitations of the analysis is the compositional structure of metagenomic data. The relative abundance of bacteria does not reflect their absolute quantity but is instead determined by the total volume of sequenced reads obtained.

## 5. Conclusions

This study suggests several observations that may reflect the complex nature of the relationship between the gut microbiota, nutritional and eating behavior patterns, and the state of the intestinal barrier in children with ASD. Already during the first year of life, microbiota formation was associated with the characteristics of feeding (BF/FF). Comparative analysis suggests that FF may be a factor associated with a less favorable microbial composition and enrichment with taxa linked to intestinal inflammation.

Food selectivity, being a key feature of eating behavior in this patient population, is accompanied by pronounced pathological shifts in the microbial profile: an increase in pro-inflammatory taxa (*Prevotella*, *Sarcina*), as well as taxa associated with neuropsychiatric symptoms (*Methanobrevibacter*, RF39). At the same time, children without food selectivity showed a compensatory increase in *Clostridia* UCG-014, which was negatively correlated with selectivity, reflecting the adaptive capacity of the microbiome in response to a more diverse diet.

Dietary interventions were associated with positive changes. Adherence to elimination diets was associated with an increase in butyrate-producing taxa (*Butyricicoccus*, UCG-003 (family Oscillospiraceae)) and a decrease in fecal calprotectin levels. The prescription of a rotational diet combined with probiotics was associated with a statistically significant decrease in fecal zonulin, which may indicate an improvement in small intestinal barrier function, as well as multidirectional changes in microbiota composition. However, despite the positive changes observed in association with dietary interventions, common features of dysbiosis persisted in all groups of children with ASD. This suggests the persistence of key microbial disturbances and points to a possible need for longer or combined approaches to correction.

Correlation analysis provided additional evidence of an association between microbiota composition and the state of the intestinal barrier: inflammation marker levels positively correlated with *Dorea* and *Streptococcus* and negatively with UCG-003 (family Oscillospiraceae), which complements existing knowledge about the gut–microbiota–brain axis in ASD.

The obtained data suggest the potential importance of considering early nutritional history for the stratification of patients with ASD and point to a possible long-term association of feeding type and eating behavior characteristics on the formation of the gut microbiota, which opens prospects for the development of personalized dietary and microbiome-modulating correction strategies.

## Figures and Tables

**Table 1:** Distribution of infant feeding types during the first year of life in the ASD1 and Control groups.

Type of Feeding	ASD1 (*n* = 96)	Control (*n* = 39)	* p * -Value
BF, abs. (%)	61 (63.5)	31 (79.4)	0.119
MF, abs. (%)	10 (10.4)	4 (10.3)	
FF, abs. (%)	25 (26.0)	4 (10.3)	

BF: breastfeeding; MF: mixed feeding; FF: formula feeding. Fisher’s exact test was used to compare categorical variables between two independent groups.

**Table 2:** Differences in microbial taxa abundance in the ASD1 Group (without diet vs. on elimination diet).

Parameter	ASD1 (Without Diet) *n* = 83, Me [IQR], %	ASD1 (on Elimination Diet) *n* = 13, Me [IQR], %	*p*-Value
Simpson index	0.97 [0.95; 0.98]	0.97 [0.96; 0.97]	0.728
Shannon index	5.96 [5.52; 6.30]	6.04 [5.58; 6.33]	0.704
ASV	218.00 [179.50; 268.00]	212.00 [184.00; 244.00]	0.764
PD	25.39 [20.42; 30.14]	22.04 [20.78; 26.69]	0.224
Chao1 index	221.58 [181.00; 269.33]	213.00 [185.00; 245.00]	0.645
* Ruminococcus *	0.99 [0.46; 2.79]	0.53 [0.39; 0.62]	0.044 *
* Butyricicoccus *	0.20 [0.10; 0.48]	0.70 [0.20; 1.00]	0.016 *
* Turicibacter *	0.05 [0.00; 0.24]	0.02 [0.00; 0.04]	0.041 *
* Erysipelotrichaceae * UCG-003	0.08 [0.01; 0.21]	0.23 [0.14; 0.35]	0.023 *
* Erysipelatoclostridium *	0.03 [0.00; 0.10]	0.00 [0.00; 0.02]	0.031 *

* *p* < 0.05; Me: median; IQR: interquartile range. *Erysipelotrichaceae* UCG-003: unclassified genus-level phylogenetic group within the family *Erysipelotrichaceae*.

**Table 3:** Paired comparison of children with ASD on a rotational diet.

Parameter	ASD1 (Rotational Diet) *n* = 47, Me [IQR], %	ASD2 (Rotational Diet) *n* = 47, Me [IQR], %	*p*
Simpson index	0.96 [0.95; 0.98]	0.97 [0.95; 0.98]	0.700
Shannon index	5.81 [5.38; 6.25]	5.86 [5.48; 6.25]	0.780
ASV	197.00 [170.00; 250.50]	209.00 [163.00; 241.00]	0.830
PD	25.45 [20.04; 31.38]	23.10 [19.52; 26.35]	0.120
Chao1 index	206.00 [173.00; 262.08]	209.38 [166.56; 249.46]	0.860
* Monoglobus *	0.43 [0.15; 1.09]	0.27 [0.07; 0.50]	0.016 *
*Sutterella*	0.01 [0.00; 0.07]	0.01 [0.00; 0.22]	0.021 *
*Butyricimonas*	0.00 [0.00; 0.12]	0.00 [0.00; 0.30]	0.038 *
* Eubacterium * ventriosum group	0.07 [0.00; 0.16]	0.02 [0.00; 0.08]	0.001 *
* Lachnospiraceae * ND3007 group	0.06 [0.00; 0.11]	0.07 [0.00; 0.26]	0.025 *

* *p* < 0.05; Me: median; IQR: interquartile range. *Eubacterium* ventriosum group: phylogenetic group within the family Lachnospiraceae; *Lachnospiraceae* ND3007 group: phylogenetic group within the family Lachnospiraceae.

**Table 4:** Significant differences in gut microbiota composition in the ASD2 group based on dietary intervention type.

Parameter	ASD2 (Rotational Diet) *n* = 47, Me [IQR], %	ASD2 (Elimination Diet) *n* = 13, Me [IQR], %	*p*-Value
Simpson index	0.97 [0.95; 0.98]	0.97 [0.95; 0.98]	0.979
Shannon index	5.86 [5.43; 6.25]	5.95 [5.68; 6.34]	0.572
ASV	209.00 [162.50; 241.00]	227.00 [172.00; 275.00]	0.240
PD	23.10 [19.82; 26.35]	25.31 [20.05; 27.59]	0.490
Chao1 index	209.38 [166.56; 249.46]	227.00 [175.75; 289.00]	0.346
*Erysipelotrichaceae* UCG-003	0.64 [0.25; 1.39]	0.13 [0.03; 0.33]	0.029 *
*Streptococcus*	0.41 [0.18; 0.90]	0.20 [0.04; 0.28]	0.022 *
*Phascolarctobacterium*	0.03 [0.00; 0.42]	0.42 [0.09; 1.01]	0.041 *
*Lachnospiraceae* UCG-001	0.00 [0.00; 0.00]	0.04 [0.00; 0.09]	0.010 *
CAG-56	0.00 [0.00; 0.06]	0.07 [0.00; 0.24]	0.018 *

* *p* < 0.05; Me: median; IQR: interquartile range. *Erysipelotrichaceae* UCG-003: phylogenetic group within the family *Erysipelotrichaceae*; CAG-352: phylogenetic group within the family Ruminococcaceae; *Lachnospiraceae* UCG-001: phylogenetic group within the family Lachnospiraceae; CAG-56: phylogenetic group within the family Lachnospiraceae.

**Table 5:** Differences in intestinal inflammation marker levels in the ASD1 Group (on diet vs. without diet).

Parameter, Me [IQR]	*n*	ASD1 (Without Diet) Me [IQR]	*n*	ASD1 (on Diet) Me [IQR]	*p*-Value
Fecal zonulin (ng/mL)	73	144.20 [94.50; 259.10]	13	98.35 [76.60; 155.65]	0.114
Fecal calprotectin (µg/g)	80	34.45 [13.33; 63.96]	13	11.97 [3.70; 28.11]	0.023 *

* *p* < 0.05; Me: median; IQR: interquartile range.

## Data Availability

The original contributions presented in this study are included in the article/Supplementary Material. Further inquiries can be directed to the corresponding author. Raw sequencing data in FASTQ format have been deposited in NCBI under BioProject ID PRJNA1459499 (http://www.ncbi.nlm.nih.gov/bioproject/1459499) (accessed on 1 May 2026).

## Supplementary Materials

The following supporting information can be downloaded at https://www.mdpi.com/article/10.3390/nu18101506/s1: Table S1: Products for the first complementary feeding of children of the ASD1 and Control groups. Table S2. Analysis of food selectivity depending on the type of feeding during the first year of life in the ASD1 group. Table S3. Sex-related differences in the relative abundance of microbial genera (median [IQR], %) in the Control and ASD1 groups. Table S4. Effect sizes (ε^2^R) for group comparisons by feeding type and food selectivity. Table S5. Differences in intestinal inflammation marker levels in the ASD1, ASD2, and control groups. Table S6. Analysis of the proportion of zonulin and calprotectin levels depending on ASD and Control.
