# Supplementary material for: Influence of Eating Behavior and Dietary Patterns on Gut Microbiota Formation in Children with Autism Spectrum Disorder
Source: Nutrients. 2026 May 8;18(10):1506. doi: 10.3390/nu18101506 (PMC13209291; doi:10.3390/nu18101506)
Supplement: Supplementary file 1 [file nutrients-18-01506-s001.zip › nutrients-4288738-supplementary.pdf]

Supplementary Materials:

Table S1. Products for the first complementary feeding of children of the ASD1 and Control groups

First feeding product	ASD1 (n=96)	Control (n=39)	p
vegetable puree, abs. (%)	70 (72.9)	26 (66.7)	0.185
porridge, abs. (%)	21 (21.9)	7 (17.9)	
fruit puree, abs. (%)	2 (2.1)	4 (10.3)	
homemade food, abs. (%)	3 (3.1)	2 (5.1)	

Table S2. Analysis of food selectivity depending on the type of feeding during the first year of life in the ASD1 group.

Parameter,	Categories	Feeding during the first year		p
		FF (n = 25)	BF (n = 61)	
Dietary pattern, n (%)	Non-selective	7 (28.0%)	25 (41.0%)	0.329
	Selective	18 (72.0%)	36 (59.0%)	

FF: formula feeding; BF: breastfeeding**.**

Table S3. Sex-related differences in the relative abundance of microbial genera (median [IQR], %) in the Control and ASD1 groups

Genus	Control (females), Me [IQR], %	Control (males), Me [IQR], %	ASD1 (females), Me [IQR], %	ASD1 (males), Me [IQR], %	Control (females) vs. Control (males)		ASD1 (females) vs. ASD1 (males)	
					P.unadj	P.adj	P.unadj	P.adj
Parasutterella	0.03 [0.00;0.20]	0.00 [0.00;0.01]	0.00 [0.00;0.04]	0.01 [0.00; 0.08]	**0,009**	0,055	0,453	0,543
Megamonas	0.00 [0.00;0.00]	0.00 [0.00;0.00]	0.00 [0.00;0.00]	0.00 [0.00; 0.00]	**0,024**	0,144	0,898	0,898
Lachnospiraceae ND3007 group	0.02 [0.00;0.07]	0.10 [0.02;0.18]	0.03 [0.00;0.08]	0.06 [0.00; 0.12]	**0,042**	0,251	0,436	0,523
Ruminococcus gnavus group	0.04 [0.00;0.15]	0.00 [0.00;0.01]	0.00 [0.00;0.01]	0.00 [0.00; 0.06]	**0,008**	**0,045**	0,540	0,648
Clostridia vadinBB60 group	0.00 [0.00;0.00]	0.00 [0.00;0.02]	0.00 [0.00;0.00]	0.01 [0.00; 0.08]	0,296	0,592	**0,045**	0,134
Lachnospiraceae UCG-010	0.00 [0.00;0.01]	0.02 [0.00;0.07]	0.00 [0.00;0.00]	0.03 [0.00; 0.11]	0,099	0,148	**0,011**	0,065

Lachnospiraceae FCS020 group	0.00 [0.00;0.06]	0.08 [0.00;0.12]	0.02 [0.00;0.04]	0.04 [0.00; 0.08]	**0,038**	0,228	0,123	0,185
Enterococcus	0.00 [0.00;0.02]	0.00 [0.00;0.00]	0.00 [0.00;0.00]	0.00 [0.00; 0.00]	**0,013**	0,078	0,825	0,825
Prevotellaceae UCG-001	0.00 [0.00;0.00]	0.00 [0.00;0.00]	0.00 [0.00;0.00]	0.00 [0.00; 0.00]	**0,042**	0,253	0,771	0,771

IQR, interquartile range; Me, median. ASD1 (males) – n = 81; ASD1 (females) – n = 15; Control (males) – n = 19; Control (females) – n = 20.

Table S4. Effect sizes (ε^2R) for group comparisons by feeding type and food selectivity

Genus	Comparison	Effect size (ε2R)
Anaerostipes	ASD1 (BF) vs. Control (BF)	0,021
Prevotella	ASD1 (BF) vs. Control (BF)	0,025
	ASD1 (non-SV) vs. Control	0,025
	ASD1 (SV) vs. Control	0,017
NK4A214 group	ASD1 (AF) vs. Control (BF)	0,030
	ASD1 (BF) vs. Control (BF)	0,021
	ASD1 (non-SV) vs. Control	0,023
	ASD1 (SV) vs. Control	0,020
Sarcina	ASD1 (BF) vs. Control (BF)	0,022
	ASD1 (non-SV) vs. Control	0,028
	ASD1 (SV) vs. Control	0,015
	ASD1(SV) vs. ASD1 (non-SV)	0,016
Methanobrevibacter	ASD1 (BF) vs. Control (BF)	0,024
	ASD1 (SV) vs. Control	0,020
RF39	ASD1 (AF) vs. Control (BF)	0,022
	ASD1 (BF) vs. Control (BF)	0,023
	ASD1 (non-SV) vs. Control	0,034
	ASD1 (SV) vs. Control	0,017
	ASD1(SV) vs. ASD1 (non-SV)	0,021
Odoribacter	ASD1 (AF) vs. Control (BF)	0,022
Family XIII AD3011 group	ASD1 (AF) vs. Control (BF)	0,028
	ASD1 (AF) vs. ASD1 (BF)	0,020
Dialister	ASD1 (non-SV) vs. Control	0,018
Clostridia UCG-014	ASD1 (non-SV) vs. Control	0,024
	ASD1(SV) vs. ASD1 (non-SV)	0,024
Clostridium sensu stricto 1	ASD1 (non-SV) vs. Control	0,018
	ASD1(SV) vs. ASD1 (non-SV)	0,017
UCG-005	ASD1 (non-SV) vs. Control	0,019

Table S5. Differences in intestinal inflammation marker levels in the ASD1, ASD2, and control groups**.**

Parameter, Me [IQR]	n	ASD1	n	ASD2	n	Control	P
		1		2		3	
Fecal zonulin (ng/mL)	86	138,40 [87,06;241,41]	44	106,55 [68,46;175,78]	22	116,33 [90,58;201,16]	p_{1-2}=0,105<br>p_{1-3}=0,358<br>p_{2-3}=0,677
Fecal calprotectin (μg/g)	93	28,11 [11,55;59,77]	57	29,17 [16,79;63,57]	38	37,52 [14,76;63,60]	p_{1-2} =0,743<br>p_{1-3} =0,865<br>p_{2-3} =0,714

IQR, interquartile range; Me, median.

Table S6. Analysis of the proportion of zonulin and calprotectin levels depending on ASD and Control**.**

Parameter	Level	ASD1 n (%)	ASD2 n (%)	Control n (%)	p
Fecal zonulin	<83.15 ng/mL	19 (22.1)	16 (36.4)	5 (22.7)	0.248
	83.15 –110.0 ng/mL	9 (10.5)	7 (15.9)	4 (18.2)	
	>110.0 ng/mL	58 (67.4)	21 (47.7)	13 (59.1)	
Fecal calprotectin	<80 μg/g	76 (81.7)	49 (86.0)	31 (81.6)	0.428
	80–160 μg/g	8 (8.6)	7 (12.3)	4 (10.5)	
	>160 μg/g	9 (9.7)	1 (1.8)	3 (7.9)	
